# Supplementary material for: Single‐molecule tracking of perfringolysin O assembly and membrane insertion uncoupling
Source: FEBS J. 2022 Sep 19;290(2):428–41. doi: 10.1111/febs.16596 (PMC10086847; doi:10.1111/febs.16596)
Supplement: Supplementary file 1 — Movie S1. PFO assembly. Movie S2. PFO plasticity [file FEBS-290-428-s001.pdf]

FEBS PRESS
science publishing by scientists
WILEY
The FEBS Journal
www.febsjournal.org

Single-molecule tracking of perfringolysin O assembly and membrane insertion uncoupling

Michael J. T. Senior, Carina Monico, Eve E. Weatherill, Robert J. Gilbert, Alejandro P. Heuck and Mark I. Wallace

DOI: 10.1111/febs.16596

Single molecule tracking the uncoupling of assembly and membrane insertion in Perfringolysin O: Supplementary Information

Michael J T Senior,[†] Carina Monico,[‡,†] Eve E Weatherill,[‡,†] Robert J Gilbert,[¶] Alejandro P Heuck,[§] and Mark I Wallace[*,‡]

†*Chemistry Research Laboratory, Department of Chemistry, University of Oxford, Oxford OX1 3TA, United Kingdom.*

‡*Department of Chemistry, King's College London, London SE1 1DB, United Kingdom.*

¶*Division of Structural Biology, Wellcome Centre for Human Genetics, University of Oxford, Roosevelt Drive, Oxford OX3 7BN, United Kingdom.*

§*Departments of Biochemistry and Molecular Biology, University of Massachusetts, Amherst, MA, USA.*

E-mail: mark.wallace@kcl.ac.uk

Supplementary Movies

Movie S1: PFO assembly

Movie of PFO assembly on a DIB by single-molecule Total Internal Reflection Fluorescence microscopy. Video starts 70 s after injection of PFO in the droplet and continues for 90 s, corresponding to the data in Fig.1B. Images are false-coloured. Exposure time 60 ms per frame and the laser power incident at the back aperture of the objective lens was 0.25 mW.

Movie S2: PFO plasticity

Example PFO plasticity event during assembly corresponding to the data in Fig.1B. Again PFO assembly was tracked on a DIB by single-molecule Total Internal Reflection Fluorescence microscopy. Exposure time 60 ms per frame and the laser power incident at the back aperture of the objective lens was 0.25 mW.
